# The impact of delays to admission from the emergency department on inpatient outcomes

**DOI:** 10.1186/1471-227X-10-16

**Published:** 2010-07-09

**Authors:** Qing Huang, Amardeep Thind, Jonathan F Dreyer, Gregory S Zaric

**Affiliations:** 1 Department of Epidemiology and Biostatistics, Schulich School of Medicine and Dentistry, University of Western Ontario, Canada; 2 Department of Family Medicine, Schulich School of Medicine and Dentistry University of Western Ontario, Canada; 3 Division of Emergency Medicine, Schulich School of Medicine and Dentistry, University of Western Ontario, Canada; 4 Richard Ivey School of Business, University of Western Ontario, Canada

## Abstract

**Background:**

We sought to determine the impact of delays to admission from the Emergency Department (ED) on inpatient length of stay (LOS), and IP cost.

**Methods:**

We conducted a retrospective analysis of 13,460 adult (≥ 18 yrs) ED visits between April 1 2006 and March 30 2007 at a tertiary care teaching hospital with two ED sites in which the mode of disposition was admission to ICU, surgery or inpatient wards. We defined ED Admission Delay as ED time to decision to admit > 12 hours. The primary outcomes were IP LOS, and total IP cost.

**Results:**

Approximately 11.6% (n = 1558) of admitted patients experienced admission delay. In multivariate analysis we found that admission delay was associated with 12.4% longer IP LOS (95% CI 6.6% - 18.5%) and 11.0% greater total IP cost (6.0% - 16.4%). We estimated the cumulative impact of delay on all delayed patients as an additional 2,183 inpatient days and an increase in IP cost of $2,109,173 at the study institution.

**Conclusions:**

Delays to admission from the ED are associated with increased IP LOS and IP cost. Improving patient flow through the ED may reduce hospital costs and improve quality of care. There may be a business case for investments to reduce emergency department admission delays.

## Background

Emergency Department ED overcrowding and delays in ED throughput have several important consequences, such as boarding of admitted patients in the ED, longer hospital stays, the inability of patients to gain access to appropriate hospital beds, lost opportunities to treat patients due to ambulance diversion, and "left without being seen" (LWBS) patients [1-6].

Current research suggests that factors external to the ED, such as hospital bed availability, laboratory turnaround, specialist consultation availability and elective surgery schedules may be more important in determining ED throughput than internal bottlenecks such as ED staff availability and bed shortages [2-4]. The 2001 position statement on ED Overcrowding by the Canadian Association of Emergency Physicians stated that hospital overcrowding was the primary cause of ED overcrowding [7]. That is, patients who should be admitted are held (boarded) in the ED because there are no hospital beds available, and this in turn uses ED resources and prevents other patients from being treated in a timely manner in the ED. This position has been echoed by professional bodies in Australia, the USA and the UK [8-10]. In addition to the potential health impact of admission delays, there may be an economic impact [11-13].

Admission through the ED accounts for a sizable portion of all admissions to surgery and inpatient wards [6]. However, there is limited evidence on the health or economic impact of emergency department admission delays in Canada. We sought to determine the impact of emergency department admission delays on two outcomes: inpatient (IP) LOS and total IP cost.

## Methods

### Study design and patient population

This was a secondary analysis using data from London Health Sciences Centre, a large multisite acute-care teaching hospital in Ontario, Canada with two adult EDs. The data was contained in three administrative databases: The National Ambulatory Care Reporting System (NACRS), which captures information on ED visits; the Discharge Abstract Database (DAD), which stores information on inpatient stays; and the hospital's case costing database, which records all resources consumed by patients during their hospital visits.

Eligible patients were all persons ≥ 18 years of age who presented to either of the EDs between April 1 2006 and March 30 2007 and who were subsequently admitted to the operating room (OR), ICU, or an inpatient ward. This patient population was selected by identifying patient IDs that were present in both the NACRS and the DAD for the same hospital encounter. Records were excluded when there were linking algorithm errors, unmatched ED or hospital stays, or a negative LOS for either the ED or the inpatient stay. Clinical information was obtained from the available data fields in the NACRS and the DAD. Cost information was obtained by linking this cohort with the case costing database. All costs are in 2006 Canadian dollars.

This study was exempted from ethics approval by the institutional ethics review board because the study used only secondary data, the data extraction and linkage was performed by staff at the hospital case costing group, and the data was stripped of patient identifiers before being given to the research team.

### Variable Definitions

**Figure 1:**
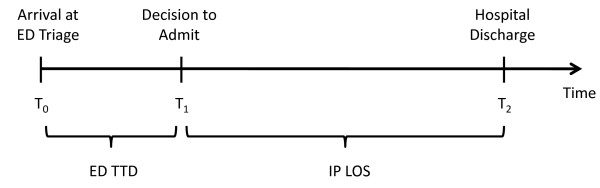
**Timeline of hospital treatment divided into ED episode and in-patient episode of care**.

We defined three time points for each patient encounter (Figure 1): time of ED triage assessment (T_0_); time of decision to admit (T_1_); and time of discharge (T_2_). All three times were recorded to include the date and time in hours and minutes. The time of decision to admit (T_1_) is the time that the admission order is written by the admitting service and is extracted by chart reviews. Pre-admission ED time to decision to admit (TTD) was the time period between arrival at ED triage and decision to admit (i.e., T_1_-T_0_). We defined delay as a binary variable taking the value 1 if ED TTD > 12 hours and 0 otherwise. We defined delay this way for two reasons. First, previous literature on this topic has used a dichotomous definition of delay, typically defining delay to occur if ED LOS > 8 hours [3,5,6,14]. Second, we believe that it would be unlikely that there would be a 12 hour delay in ED TTD due to patient complexity alone, and that a delay of this magnitude would be caused, at least in part, by system factors.

Our first outcome, IP LOS, was the time between T_1 _and T_2_. Our second outcome, total IP cost, was the cumulative cost incurred from T_1 _to T_2_.

In multivariate analysis we included the following covariates: age, age^2^, gender (0 = male 1 = female), arrival by ambulance (0 = no 1 = yes), admission to ICU or surgery (0 = general wards 1 = ICU or surgery), case mix group (CMG), ED triage category, and site of ED. We included age to account for the possibility that older patients may be more complex and require more time to treat. We included age^2 ^as a mathematical means to account for the possibility that the trend in age is non-linear (i.e., the increase in complexity associated with a 1-year increase in age would be greater among older patients than among younger patients). We included CMGs, which categorize patients into clinically homogenous groups, to adjust for severity of illness and case complexity. We included a separate binary variable for each of 350 groups in the data set. CMGs for inpatients are determined by the Health Records department at the study institution. An algorithm provided to Canadian hospitals by the Canadian Institute for Health Information (CIHI) is used to abstract relevant information from each patient's chart in order to assign a CMG. ED triage categories were included to adjust for initial acuity. The ED triage categories were defined according to the 5-level Canadian Triage and Acuity Scale (CTAS), which groups patients as follows: CTAS 1 - Resuscitation, CTAS 2 - Emergent, CTAS 3 - Urgent, CTAS 4 - Less Urgent and CTAS 5 - Non-Urgent. The specific site of an ED visit was included to adjust for site level characteristics.

### Statistical Analysis

We performed univariate analysis which included the construction of Kaplan-Meier survival curves. In multivariate analysis we used natural logarithm transformations of IP LOS and IP cost to account for the skewed distributions of LOS and cost.

## Results

### Study Population

The initial dataset contained 10,975 unique patients, who made 13,648 visits to the ED that resulted in hospital admissions (1.24 visits per patient). We excluded 188 visits with data linking algorithm errors, unmatched ED or hospital stays, or negative time intervals. The final data set contained 10,847 unique patients who made 13,460 visits to the ED that resulted in hospital admissions (Table 1). The mean age was 62.6 years and the sample contained approximately equal numbers of males and females. Approximately 11.6% (n = 1558) of patients experienced admission delay. Of those admitted, 14% were admitted to ICU or surgery. A higher proportion of non-delayed patients were admitted to ICU or surgery compared to patients in the delayed group (15% versus 7%; p < .0001). After completion of hospital treatment, 74% were discharged home, 17% were discharged to destinations with some level of additional care and 8.7% of patients died in hospital.

**Table 1:** Characteristics of Emergency Department patients who were admitted to the hospital, by presence or absence of admission delay.†^¶^

Characteristic (n,% *)	All	No Delay	Delay	p value
	N = 13460	N = 11902	N = 1558	
Unique patients	10846			
Age, yr (mean, SD)	62.6 (20)	62.3 (20)	64.4 (19.7)	p < .0001
Sex, male	5390 (50)	6082 (51)	684 (44)	p < .0001
**ED EPISODE**				
**Mode of arrival to ED**				
Ambulance	7310 (54)	6397 (54)	810 (52)	0.19
Ambulatory ("Walk-in")	6067 (45)			
Not available	78 (0.5)			
**ED Triage Category by CTAS Level**				
CTAS level 1 - Resuscitation	623 (4.6)	606 (5.1)	17 (1.1)	p = 0.04
CTAS level 2 - Emergent	3898 (29)	3540 (29.7)	358 (2.7)	
CTAS level 3 - Urgent	7613 (56.6)	6654 (55.9)	959 (61.6)	
CTAS level 4 - Less Urgent (Semi-Urgent)	1307 (9.7)	1086 (9.1)	221 (14.2)	
CTAS level 5 - Non-Urgent	19 (0.14)	16 (0.13)	3 (0.19)	
**Total cost of Emergency Department stay **(mean, SD)	1027 (760)	948 (701)	1,631 (914)	p < .0001
**ED LOS**, minutes **(**mean, SD)	419 (307)	336 (174)	1059 (348)	p < .0001
**ICU or surgery admission**	1936 (14)	1827 (15)	109 (7)	p < .0001
**IN-PATIENT EPISODE**				
**Destination after hospital stay**				
Transfer to in-patient facility	372 (3)	355 (3)	17 (1)	p < .0001
Transfer to long term care	1780 (13)	1530 (13)	250 (16)	
Transfer to nursing home	14 (0.10)	14 (0.11)	1 (0.06)	
Discharged home with support	2385 (18)	2068 (17)	317 (20)	
Discharged home	7598 (56)	6756 (57)	842 (54)	
Left against medical advice	142 (1)	125 (1)	17 (1)	
Died in hospital	1169 (9)	1055 (9)	114 (7)	
**Hospital length of stay**, days (mean, SD)	8.8 (15)	8.5 (13.9)	11.3 (20.7)	p < .0001
**Total cost of hospital stay **(mean, SD)	11,064 (13,917)	10,902 (19,991)	12,307 (24,438)	p = 0.03
**Mortality, %**	1169 (9)	1055 (9)	114 (7)	p = 0.04

SD = standard deviation.

† As defined by pre-admission length of stay of greater than 12 hours in the Emergency Department†

* Unless stated otherwise

**Figure 2:**
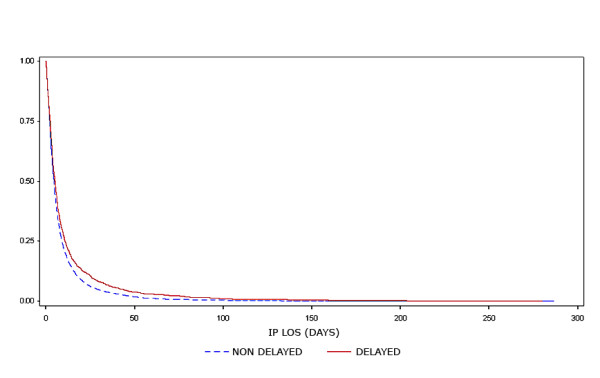
**Unadjusted Kaplan-Meier survival curve comparing hospital length of stay of delayed versus non-delayed patients**.

The average ED TTD was 419 minutes (median 359.5, IQR 215 - 535). The average ED TTD differed by group and was 336 minutes (median = 325) among those who experienced no delay and 1059 minutes (median = 940) among those who were delayed. The average IP LOS was 8.8 days (median 4.6, IQR 2.2 - 9.2) and also differed by group, with an average of 8.5 days in the non-delay group (median = 4.6) and 11.3 days in the delay group (median = 5.2). A Kaplan-Meier survival curve (Figure 2) illustrates the difference in IP LOS between the delay group and the non-delay group. The average IP cost was $11,064 (median $5,256, IQR $2,683 - $11,344). In univariate analysis the difference in average cost was significant (p = 0.04), $10,902 in the non-delay group (median $5,238) compared to $12,307 (median $5,449) in the delayed group.

Among the 1936 patients who were admitted to ICU or surgery, 109 (5.6%) experienced delay. As in the previous case, the IP LOS was longer among delayed patients: 7.9 days for delayed patients versus 8.3 days for non-delayed patients. However, unlike the previous case, the cost was higher among non-delayed patients: $16,167 among non-delayed patients versus $13,075 among delayed patients.

### Multivariate Analysis

#### IP LOS

The fitted multivariate regression model showed that delayed patients have on average 12.4% (95% confidence interval [CI] 6.6% - 18.5%) longer IP LOS compared to patients who were not delayed (p < .0001), adjusting for age, sex, ED triage urgency, arrival by ambulance, ICU admission, site of ED, and CMG (Table 2). This corresponds to an absolute increase in IP LOS of approximately 1.2 days (11.3 - 11.3/exp(.117) = 1.2).

**Table 2:** Results of the multivariate models for hospital length of stay and total hospital cost

Predictor	Hospital Length of Stay			Total Hospital Cost		
	
	Coefficient	Impact of Factor (Percent Change on Outcome†)	p value	Coefficient	Impact of Factor (Percent Change on Outcome†)	p value
Admission Delay > 12 hrs (no = 0, yes = 1)	0.117	12.4% (6.6 - 18.5)	< .0001	0.104	11.0% (6.0 - 16.4)	< .0001
CTAS 1 - Resuscitation*	-0.084	-8.0% (-16.2 - 0.91)	0.0768	0.32	37.6% (26.9 - 49.3)	< .0001
CTAS 2 - Emergent*	0.0044	0.44% (-3.7 - 4.7)	0.84	0.079	8.2% (4.3 - 12.3)	< .0001
CTAS 4 - Less Urgent*	-0.033	-3.3% (-8.9 - 2.7)	0.27	-0.055	-5.3% (-10.2 - -0.21)	0.04
CTAS 5 - Non-Urgent*	-0.41	-33.8 (-57.6 - 3.9)	0.07	-0.51	-40% (-59.4 - -10.9)	0.011
Arrival by Ambulance (no = 0, yes = 1)	0.12	12.9% (8.7 - 17.3)	< .0001	0.168	18.3% (14.4 - 22.3)	< .0001
Sex (male = 0, female = 1)	-0.077	-7.4% ( -10.6 - -4.1)	< .0001	-0.041	-4.0% (-6.9 - -1.0)	0.009
Admit to ICU or OR ( no = 0, yes = 1)	-156	-14.5% (-19.1 - -9.6)	< .0001	0.19	21% (15.2 - 27.0)	< .0001
Age (years)	0.011	1.1% (1.0 - 1.2)	< .0001	0.0088	0.9% (0.8 - 1.0)	< .0001
Age2 (years2)	0.0000072	0.00072% (-0.004 - 0.005)	0.75	-0.000028	-0.003% (-0.007 - 0.001)	0.17
Site of ED	0.037	3.8% (0.2 -7.6)	0.04	0.038	3.9% (0.7 - 7.2)	0.016
CMGs 001-901	Included in model to adjust for the effects of disease conditions; individual values are not shown					

* CTAS 3 -Urgent is the reference group

† Converted from the lognormal model to percentage impact by taking Exp(coefficient value); e.g., Exp(0.117) = 1.124, which corresponds to a 12.4% increase in hospital LOS associated with admission delay > 12 hours.

#### IP Cost

The fitted multivariate model for total hospital cost showed that admission-delayed patients have on average 11.0% (95% CI: 6.0% - 16.4%) higher IP cost compared to patients who were not delayed (p < .0001), adjusting for age, sex, ED triage urgency, arrival by ambulance, ICU admission, site of ED, and CMG (Table 2). This corresponds to an absolute difference in IP cost of approximately $1216 (12,307-12,307/exp(.104) = 1216).

### Patients Admitted to ICU or Surgery

We fitted multivariate regression models for IP LOS and IP cost using only those patients admitted to ICU or surgery (excluding CMG as a covariate). In both cases the ED TTD variable was not significant (p > 0.1).

### Cumulative Impact of Delay

We estimated the cumulative impact of these delays on the study hospital. IP LOS was 11.3 days among delayed patients, and delay was associated with a 12.4% increase in IP LOS. Thus, the cumulative impact of delay was 1558 patients × 11.3 days × 12.4% = 2183 additional hospital days. Using the 95% confidence intervals the excess hospital days due to admission delay could be as low as 6.6% (1162 days) or as high as 18.5% (3257 days).

IP cost was $12,307 among delayed patients and delay was associated with an 11% increase in IP cost. Thus, the cumulative impact of delay was 1558 patients × $12,307 × 11% = $2,109,173, or approximately $1354 per admitted patient who experiences delay. The 95% confidence interval for increased costs ranges from $1,150,458 to $3,144,586.

## Discussion

This is the first study that we know of to estimate the impact of delays to admission from the ED on inpatient hospital outcomes in Canada. In multivariate analysis we found that patients who experienced admission delay in the ED had 12.4% longer IP LOS and incurred 11.0% higher IP costs compared to patients who were not delayed. This association is important because approximately 11% of admissions from the ED experienced delay and the cumulative effects of these delays on cost and IP LOS can be substantial.

Our analysis suggest that there may be a purely financial "business case" for investments that improve ED throughput and reduce delays. That is, there may be system-wide saving associated with investments targeted to improving ED throughput. In our sample the cumulative effect of delay for the 1558 patients who experienced delay was 2183 extra hospital days and $2,109,173 in additional hospital cost corresponding to approximately $1354 per admitted patient who experiences delay. This amount is relatively small compared to the total hospital budget but relatively large compared to the marginal cost of interventions that could reduce delay, such as opening another inpatient bed or funding another specialist.

ED delay can be due to both patient complexity and true ED delay on the part of the care delivery system. We adjusted for initial acuity using CTAS score and by considering whether admission was to surgery or ICU. In addition, we adjusted for final complexity using most responsible diagnosis and age. Finally, we used a rough measure of delay, ED TTD > 12 hours. We believe that it is unlikely that a patient would remain in the ED for more than 12 hours due to patient factors alone. In additional analyses we investigated other definitions of "Delay" and we found a dose-response relationship - patients with longer delays in ED TTD experienced greater increases in IP LOS and IP cost [15].

The association between ED LOS and hospital LOS has been studied by others. Richardson used ED LOS > 8 hours to define admission delay and found that on average, delayed patients stayed 6.5 hours longer in the ED and 0.8 days longer as inpatients than non-delayed patients. The estimated cumulative impact at the study site was 700 bed-days per year [5]. Liew et al studied 17,954 admissions from the ED in three Australian hospitals from July 2000 to June 2001 [6]. They found that prolonged ED LOS was associated with excess inpatient LOS in a "dose-dependent" relationship. Compared to patients with ED LOS < 8 hours, patients with ED LOS of 8-12 hours were approximately 20% more likely to have longer inpatient LOS, and patients with ED LOS > 12 hours were 50% more likely to have longer inpatient LOS.

We are aware of two other attempts to investigate the cumulative financial impact of delay. In the first, Krochmal et al [13] conducted a retrospective analysis of 26,020 admissions from a single ED in the US over 3 years. They compared IP LOS between those patients who were still present in the ED at midnight and those who were admitted before midnight each day. The authors estimated a cost per inpatient day of $800 by dividing the total funding by the total number of patient days. This resulted in an estimate of the cumulative impact of $6.8 M and 8455 excess inpatient days. However, there are some limitations to their analysis: the use of ED census at midnight as an indicator of delay may result in patients with relatively short ED stays being classified as delayed; the cost of $800 per day was for an average patient rather than being patient specific; and only Medicare patients were included in the analysis.

In the second investigations, Falvo and colleagues reported in two separate papers on the cumulative financial impact of delay used data from 62,588 patient records collected over a 12 month period at a hospital in Pennsylvania [11,12]. In the first paper they estimated that the cumulative impact of ambulance diversion and "left without being seen" patients was $2.9 M [12]. In the second they estimated that 29% of admitted patients experienced delays in the ED, and that this translated to 10,397 lost treatment hours valued at $3.9 M [11].

## Limitations

We were unable to capture the actual time that patients were sent to inpatient units as this information was not captured in the data bases used for the analysis. Thus, our analysis may underestimate the true impact on ED resources. We used a retrospective study design that does not allow us to isolate the cause of admission delays. Thus, we can only speculate as to whether or not the delay was due to lack of availability of hospital beds or other barriers to treatment or assessment. Prolonged IP LOS may also be caused by downstream problems including discharge difficulties, such as lack of rehabilitation beds or difficulties coordinating outpatient care [5]. We were not able to assess whether this was a contributing factor to longer hospital LOS and higher costs.

We used case mix groups and location of admission to adjust for patient acuity. However, patients in the same group may still differ in clinically important ways which would affect their IP LOS and IP cost. Although we accounted for initial acuity and final complexity through triage severity, admission to ICU wards, most responsible diagnosis and age, we may not have controlled for patient complexity delay completely. However, we believe that a wait of > 12 hrs would be unlikely to be the result of patient complexity delay alone. Finally, our analysis is based on a single academic hospital and the results may not be generalizable to other settings.

## Conclusions

Our study shows that among patients admitted to the hospital from the ED, ED LOS > 12 hours is associated with 12.4% longer IP LOS and 11% greater IP cost. The cumulative effect of delay on the 1558 patients who experienced delay was an additional 2,183 hospital days and $2,109,173 in incremental cost. These figures suggest that there may be a business case for interventions that improve ED flow and reduce admission delay.

## Abbreviations

(CMG): Case Mix Group; (CTAS): Canadian Triage and Acuity Scale; (ED): Emergency Department; (ICU): Intensive Care Unit; (IP): Inpatient; (LOS): Length of Stay; (TTD): Time To Decision to Admit.

## Competing interests

During the past 5 years JFD has been an emergency physician at the study institution. As such he is an independent medical practitioner and is not paid by this organization.

## Authors' contributions

QH and GSZ were responsible for the study conception and design and acquisition of data. All authors contributed to the analysis and interpretation of data; were involved in drafting the manuscript or revising it critically for important intellectual content; and have given approval to the final manuscript.

## Pre-publication history

The pre-publication history for this paper can be accessed here:

http://www.biomedcentral.com/1471-227X/10/16/prepub
